# A duct-to-mucosa pancreaticojejunostomy for small main pancreatic duct and soft pancreas in minimally invasive pancreaticoduodenectomy

**DOI:** 10.1007/s00464-022-09830-6

**Published:** 2023-01-09

**Authors:** Anbang Zhao, Qian Zhu, Xian Qin, Kunlei Wang, Kai Tan, Zhicheng Liu, Wenjing Song, Qian Cheng, Xinyin Li, Zhinan Chen, Zhisu Liu, Yufeng Yuan, Zhiyong Yang

**Affiliations:** 1 Department of Hepatobiliary and Pancreatic Surgery, Zhongnan Hospital of Wuhan University, Wuhan, China; 2 Pancreatic Surgery Center, Zhongnan Hospital of Wuhan University, Wuhan, China; 3 Clinical Medicine Research Center for Minimally Invasive Procedure of Hepatobiliary & Pancreatic Diseases of Hubei Province, Hubei, China

**Keywords:** Minimally invasive pancreaticoduodenectomy, Pancreaticojejunostomy, Pancreatic fistula

## Abstract

**Background:**

Postoperative pancreatic fistula (POPF) is often associated with significant morbidity and mortality after the Whipple operation. Patient-related factors associated with POPF include soft pancreatic texture and a small main pancreatic duct (MPD). The traditional duct-to-mucosa anastomosis was modified to be easily performed. The aim of the study was to evaluate the simplified pancreaticojejunostomy (PJ) method in the prevention of POPF after minimally invasive pancreaticoduodenectomy (PD).

**Methods:**

Ninety-eight patients who underwent laparoscopic pancreaticoduodenectomy (LPD) and robotic pancreaticoduodenectomy (RPD) with a simplified PJ procedure containing only two duct-to-mucosa sutures and four penetrating-sutures to anastomose the pancreatic parenchyma and jejunal seromuscular layer in our center were retrospectively studied. Demographics and clinical short-term safety were assessed.

**Results:**

All LPD and RPD procedures were successfully performed. The median time of PJ was 17 min, and the median blood loss was 60 mL, with only one patient requiring transfusion. Four patients (4.1%) suffered from clinically relevant POPF (CR-POPF), including four grade B cases and no grade C cases. For patients with an MPD diameter of 3 mm or less, POPF was noted in two (4%) of the fifty patients, with all cases being grade B. Of the patients with a soft pancreas, only two (4.5%) patients suffered from grade B POPF. One patient (1.0%) experienced a 90-day mortality. Neither the main pancreatic diameter nor pancreatic texture had an impact on postoperative outcomes.

**Conclusions:**

Our technique is a simple, safe and efficient alternative to prevent POPF after LPD and RPD. This method is suitable for almost all pancreatic conditions, including cases with a small main pancreatic duct and soft pancreas, and has the potential to become the preferred procedure in low-volume pancreatic surgery centers.

**Graphical abstract:**

Our modified duct-to-mucosa PJ, which contains only two duct-to-mucosa sutures and four penetrating-sutures to anastomose the pancreatic parenchyma and jejunal seromuscular layer, is ideal for small MPD and soft pancreas when performing minimally invasive PD and has a low rate of POPF. PJ pancreaticojejunostomy, MPD main pancreatic diameter, PD pancreaticoduodenectomy, POPF postoperative pancreatic fistula

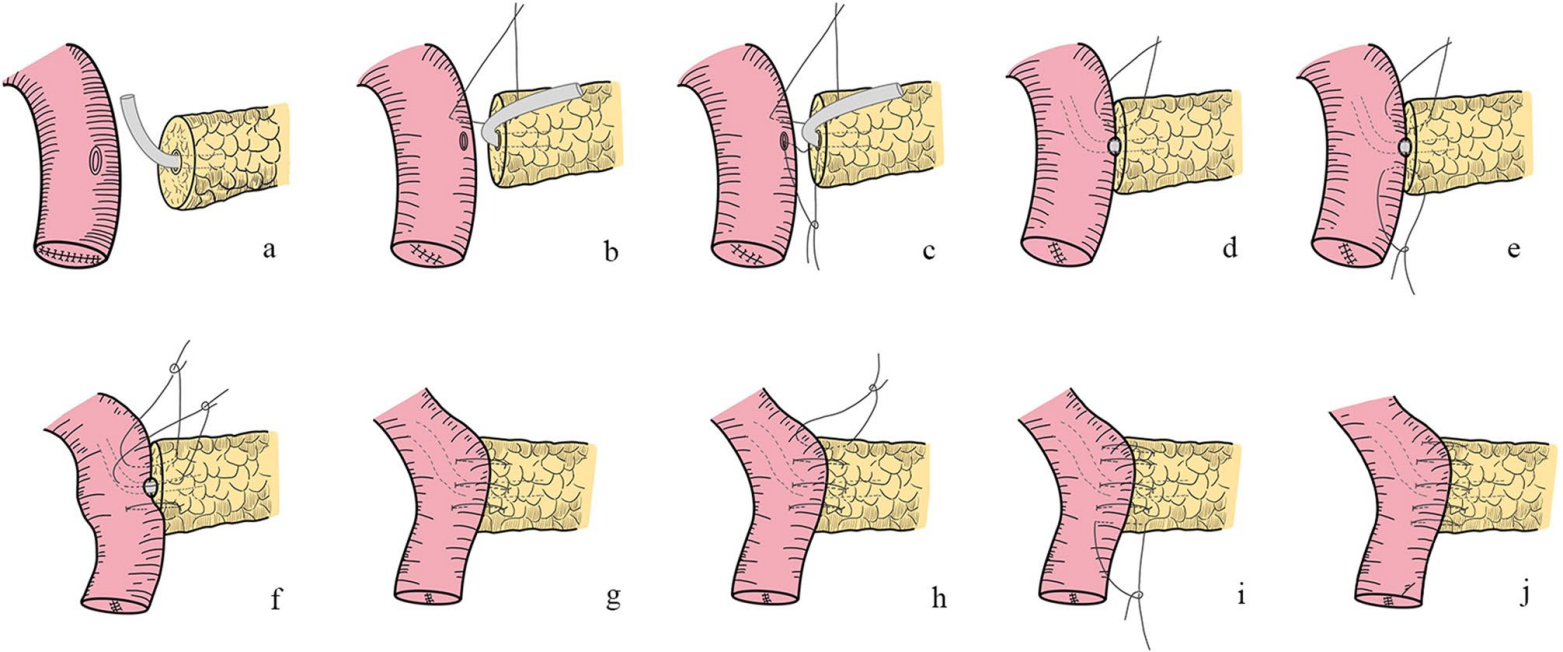

**Supplementary Information:**

The online version contains supplementary material available at 10.1007/s00464-022-09830-6.

Pancreaticoduodenectomy (PD) is the standard of therapy for patients with malignant or benign disease of the pancreatic head or periampullary region. In recent years, some doctors have proposed surgical treatment using minimally invasive procedures, including laparoscopic pancreaticoduodenectomy (LPD) and robotic pancreaticoduodenectomy (RPD), which seem to show comparable clinical outcomes, including operative time and R0 resection rate, to open surgery, despite being considered inferior in the past [1–3]. Minimally invasive PD also has unique advantages. LPD allows for smaller surgical incisions, and RPD overcomes several limitations related to LPD, such as optical vision, a steep learning curve and surgeon tremor [4–7]. The transition from open surgery to robotic PD seems to be easier compared with the transition from open surgery to LPD. However, no differences are currently found in clinically relevant parameters between the two minimally invasive approaches [8]. These two minimally invasive procedures have been used in some high-volume pancreatic surgery centers with remarkable results [9].

Postoperative pancreatic fistula (POPF) is often associated with significant morbidity and mortality after the Whipple operation [10–14]. POPF can result in intra-abdominal infection, intra-abdominal hemorrhage, prolonged hospital stays, the need for reoperation or interventional therapy, and even death.

Patient-derived factors associated with pancreatic anastomotic failure have been identified and include soft pancreatic texture, a small MPD and a poor blood supply [15–19]. To reduce the incidence and related complications associated with pancreatic fistula, numerous anastomotic techniques and pharmacologic interventions have been proposed and studied [20–25]; however, there is still no accepted standard approach for decreasing pancreatic fistula after PD.

Essential criteria in an “optimal” technique for pancreaticojejunostomy (PJ) should be associated with a low rate of significant pancreatic anastomotic failure-related complications and mortality; additionally, this technique should be easy to learn, perform and duplicate [26]. Two main methods are currently used for PJ anastomosis, including the invagination and “duct-to-mucosa” anastomosic techniques. Pancreatic penetrating-suture has gained wide acceptance in recent years because it is suitable for a soft and fragile pancreas [14, 24, 27, 28]. Penetrating-suture is not only easy to achieve but also reduces the possibility of laceration for a fragile pancreas because more pancreatic parenchyma is bundled by a single suture. In 1996, the Japanese scholar Kakita proposed a new PJ with fewer sutures that used full-thickness sutures to complete anastomosis of the pancreatic stump and jejunal loop for the first time. At the time, this technique had a very low rate of grade B + C POPF (1.2%) [29], and it became the most popular PJ technique in Japan.

Herein, we propose a duct-to-mucosa PJ technique that can be performed in LPD and RPD. The technique was developed based on the Kakita PJ and modified to be simpler and easier. This procedure requires only 6 sutures, and the clinical outcomes were excellent.

## Materials and methods

This study was approved by the Medical Ethics Committee of the Zhongnan Hospital of Wuhan University (2022113 K).

### Patient selection

**Fig. 1:**
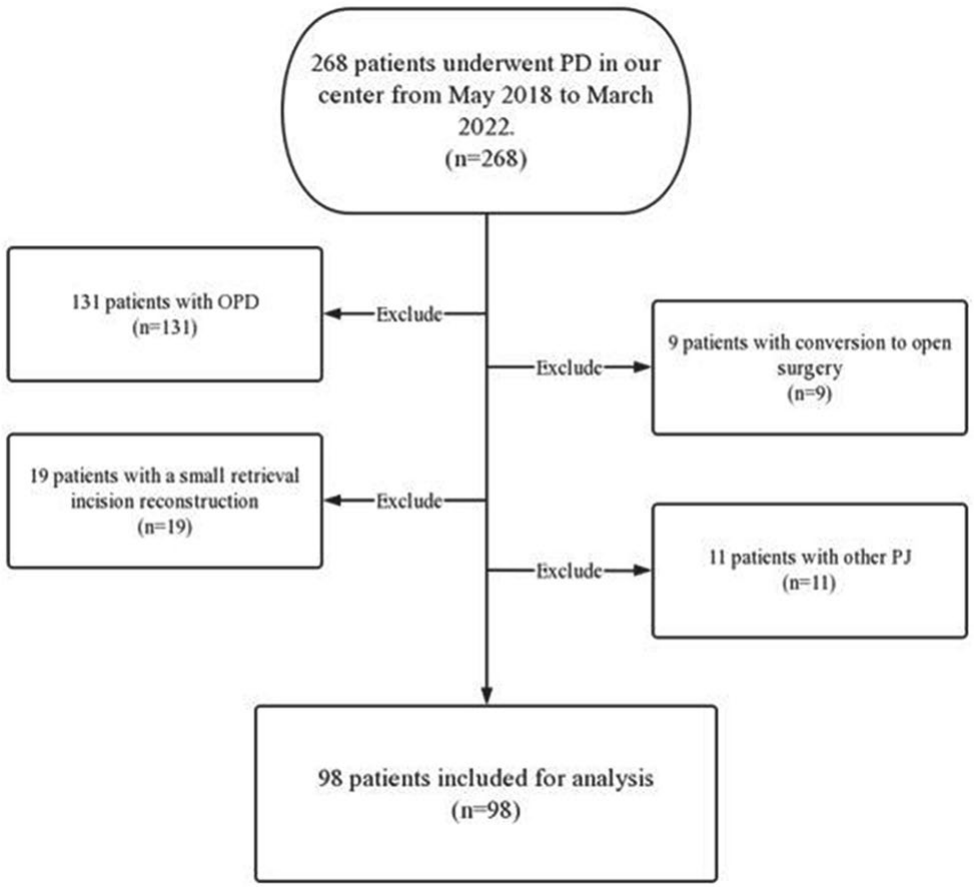
Flow diagram of inclusion and exclusion criteria

From May 2018 to March 2022, 98 consecutive patients underwent minimally invasive PD with modified Kakita PJ in our center. The selection criteria for our study included the following: (1) patients who underwent LPD or RPD and (2) patients who underwent modified Kakita PJ. The exclusion criteria were as follows: (1) patients who underwent OPD; (2) conversion to open surgery; (3) small retrieval incision reconstruction; and (4) other PJ procedures. (Fig. 1).

### Clinical information

The demographic characteristics and perioperative details were accurately collected. The diameter of the MPD was measured at the level of the pancreatic neck by computed tomography, and the pancreatic texture was evaluated by the surgeon. For postoperative outcomes, the diagnostic criteria for POPF, biliary fistula, delayed gastric emptying (DGE) and postpancreatectomy hemorrhage (PPH) were classified according to the International Study Group of Pancreatic Surgery (ISGPS) [30]. To verify that our modified Kakita PJ was effective regardless of the pancreatic conditions, we divided patients into two groups according to pancreatic texture and MPD size. Furthermore, we evaluated risk factors for anastomoses for pancreatic surgeries through the four-tier classification system by ISGPS to achieve international comparability [31].

To verify the application of modified Kakita PJ in various preoperative risk groups, we divided patients into low-risk and high-risk groups based on the benchmark case selection criteria to better evaluate the relationship between postoperative complications and preoperative risk in patients who underwent our PJ procedure [32].

### Surgeons and pancreatic surgery center

All minimally invasive PDs were performed at the Pancreatic Surgery Center, Zhongnan Hospital of Wuhan University. A total of 100–150 pancreatic surgeries are performed in our center each year, of which 58–80 are PD, and the minimally invasive PD rate is 50–60%. The main surgeon (Dr. Zhiyong Yang) had 8 years of experience with independent PD procedures (nearly 450 OPDs, 42 LPDs and 15 RPDs) prior to the study and performed nearly 90% of minimally invasive PD procedures in our center.

### Modified kakita PJ (video)

**Fig. 2:**
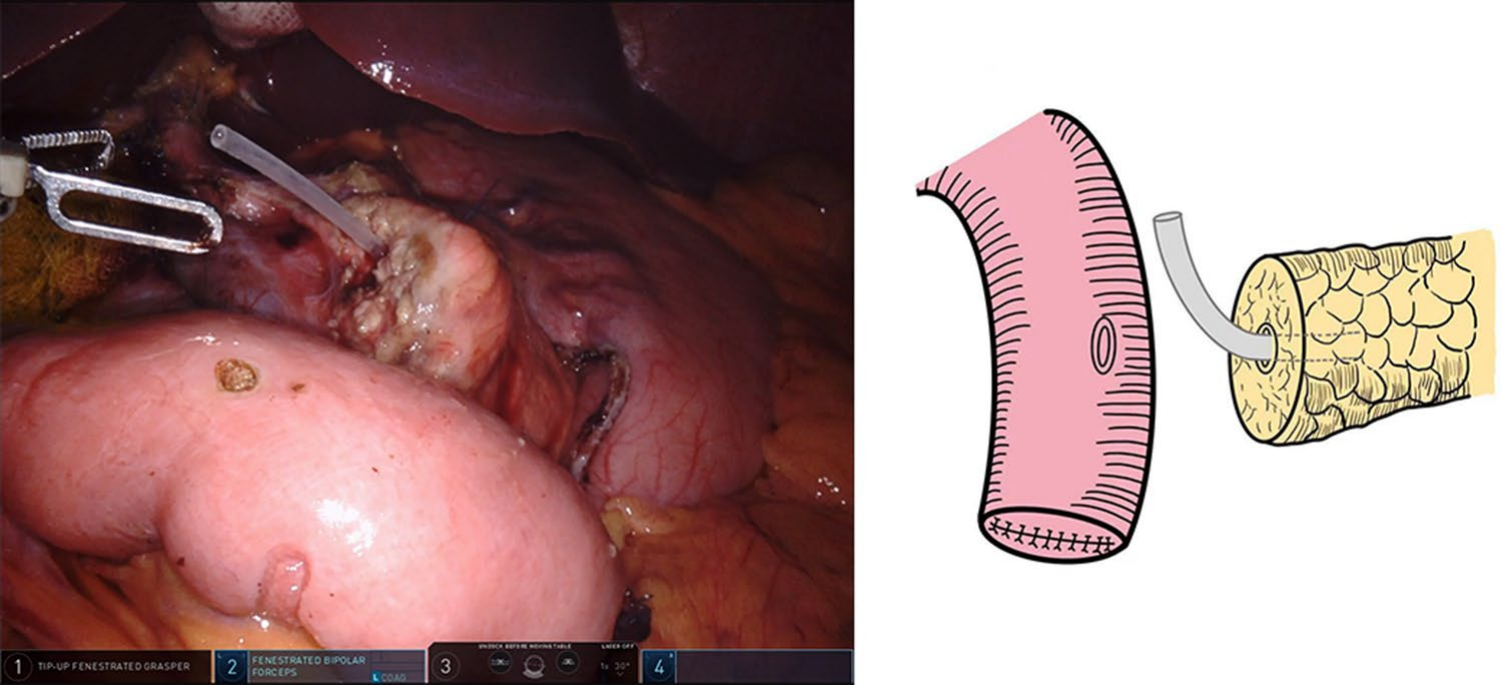
The jejunostomy was made with a full-thickness hole, and a small silicone stent was placed in the pancreatic duct

The end of the jejunal loop was closed by a stapler. A small, full-thickness opening was made on the anti-mesenteric border at approximately 6 cm to the jejunal stump. A 1–3 mm stent was inserted into the MPD remnant (Fig. 2).

**Fig. 3:**
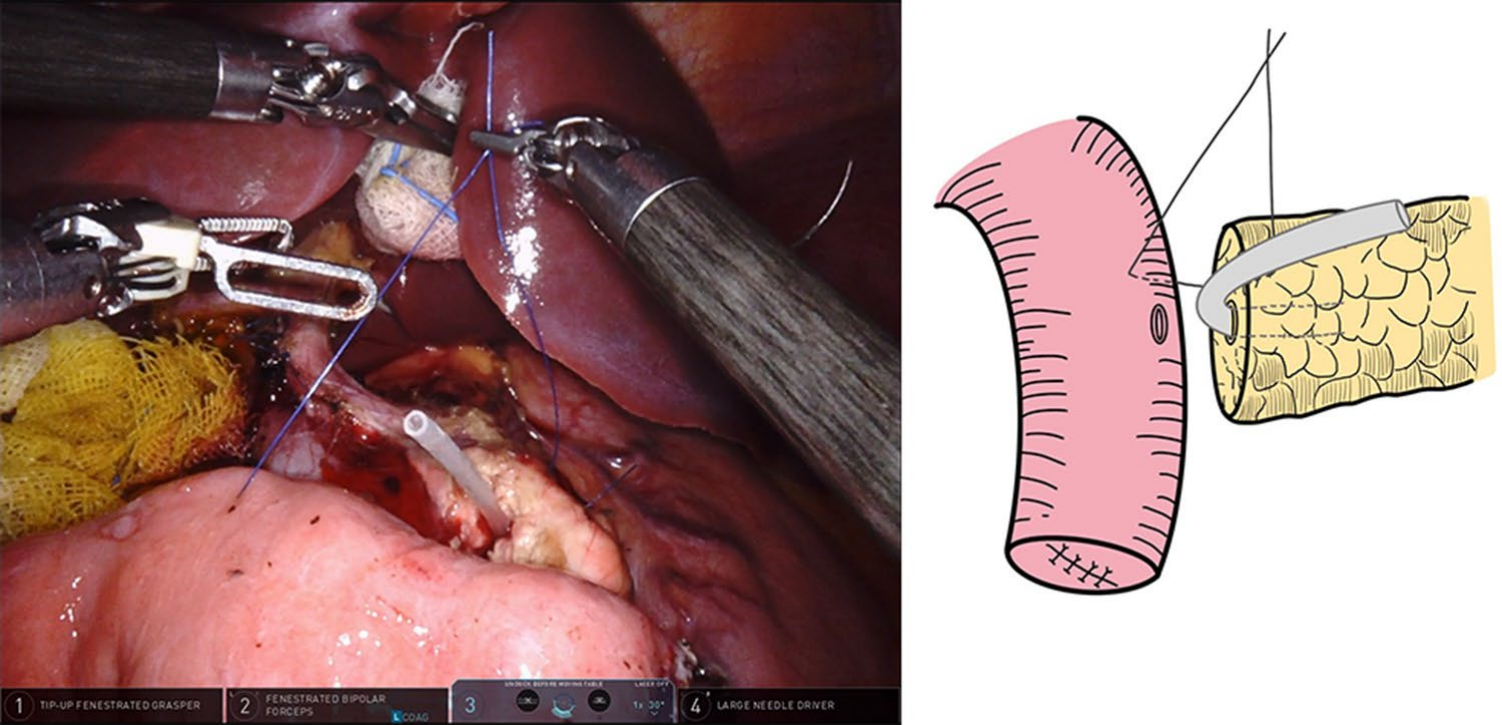
The first suture was performed to draw the pancreatic stump and jejunal wall close, which facilitated subsequent anastomosis

To anastomose the pancreatic stump and jejunal wall, needles with a 3–0 non-absorbable Prolene (Ethicon, 8842, 36 mm, 1/2c) suture were used in an end-to-side penetrating pattern. The first stitch (15 cm in length), which was close to the MPD, completely penetrated the pancreatic parenchyma from anterior to posterior and then from posterior to anterior, traversing the seromuscular layer of the jejunum wall. The edge distance was approximately 1.0 cm on the pancreatic stump, while the stitch width on the jejunum wall was slightly larger than the pancreatic stump thickness. When finished, the pancreatic stump and jejunal wall were drawn close (Fig. 3).

**Fig. 4:**
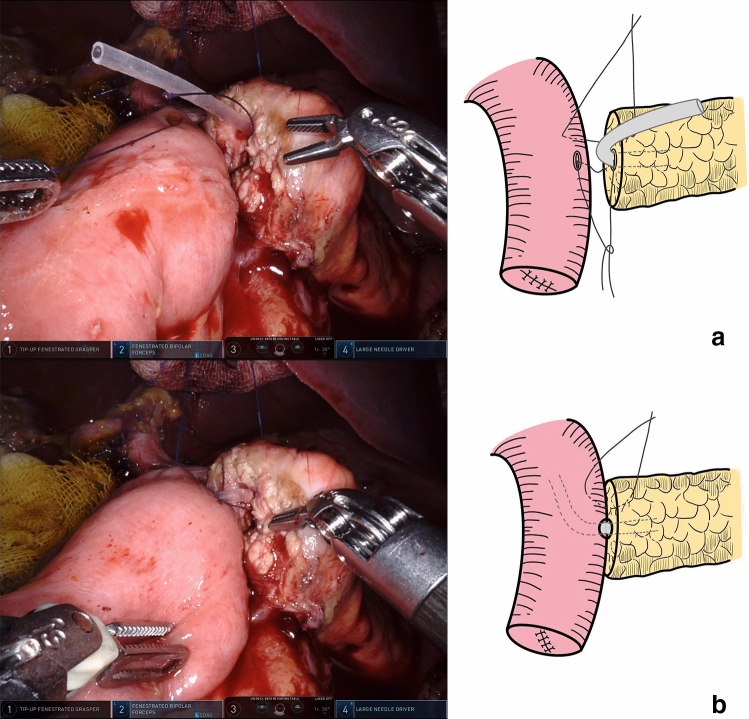
**a** The stitch penetrated the pancreas from the duct to the posterior stump and entered the jejunum wall posterior to the jejunum hole. **b** The stent was inserted into the jejunum after the stitch was knotted

Then, to anastomose the MPD and jejunal mucosa, needles with a 4–0 absorbable Vicryl (Ethicon, VCP771D, 22 mm, 1/2c) suture were used. The second stitch (12 cm in length) penetrated the pancreas from the duct to the posterior stump and entered the jejunum wall posterior to the jejunum hole. The stitch of the MPD’s posterior wall was knotted prior to stent insertion into the jejunum (Fig. 4).

**Fig. 5:**
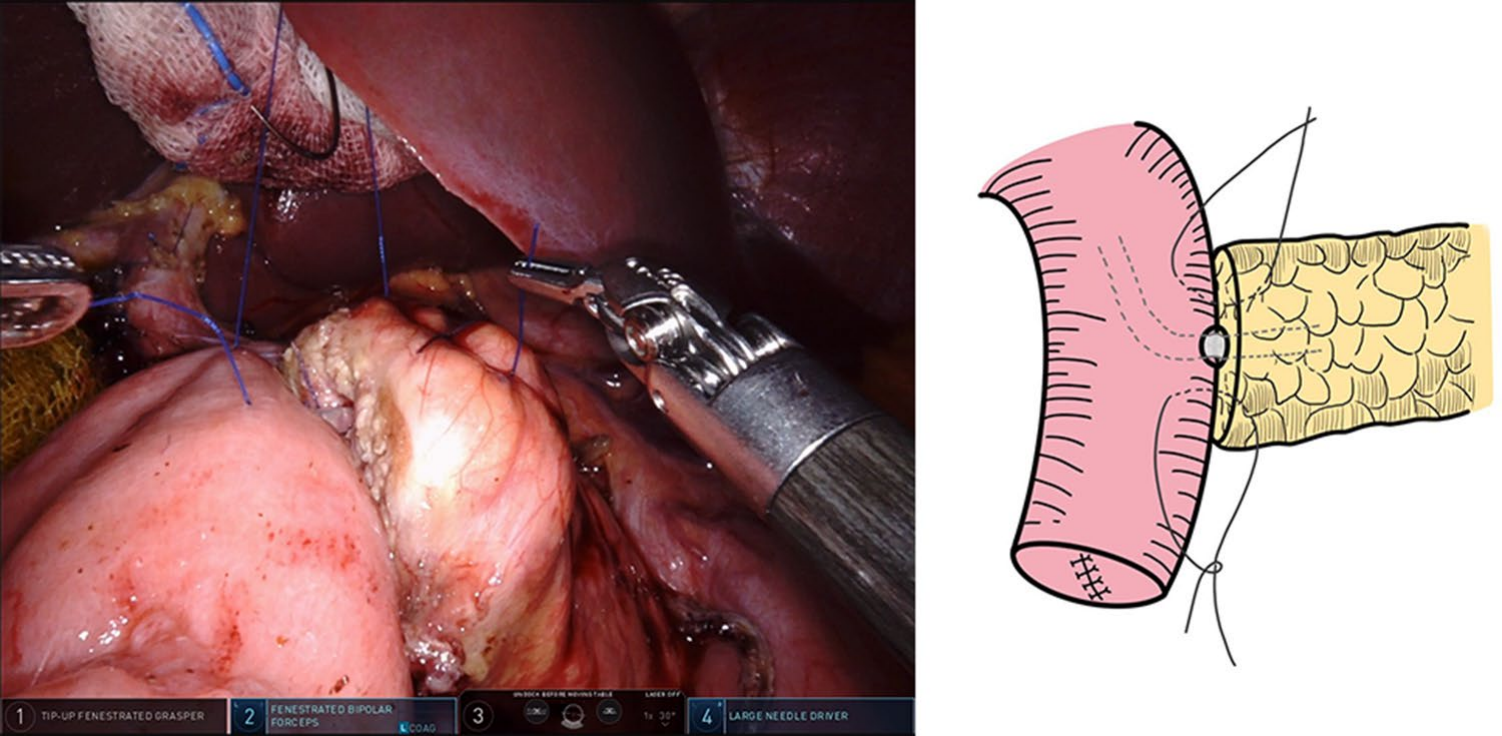
The second penetrating stitch opposite to the first stitch was used to anastomose pancreatic stump and jejunal wall

At this point, the second penetrating stitch (12 cm in length) to anastomose the pancreatic stump and jejunal wall was completed and knotted. This stitch was opposite to the first stitch and was also close to the MPD (Fig. 5).

**Fig. 6:**
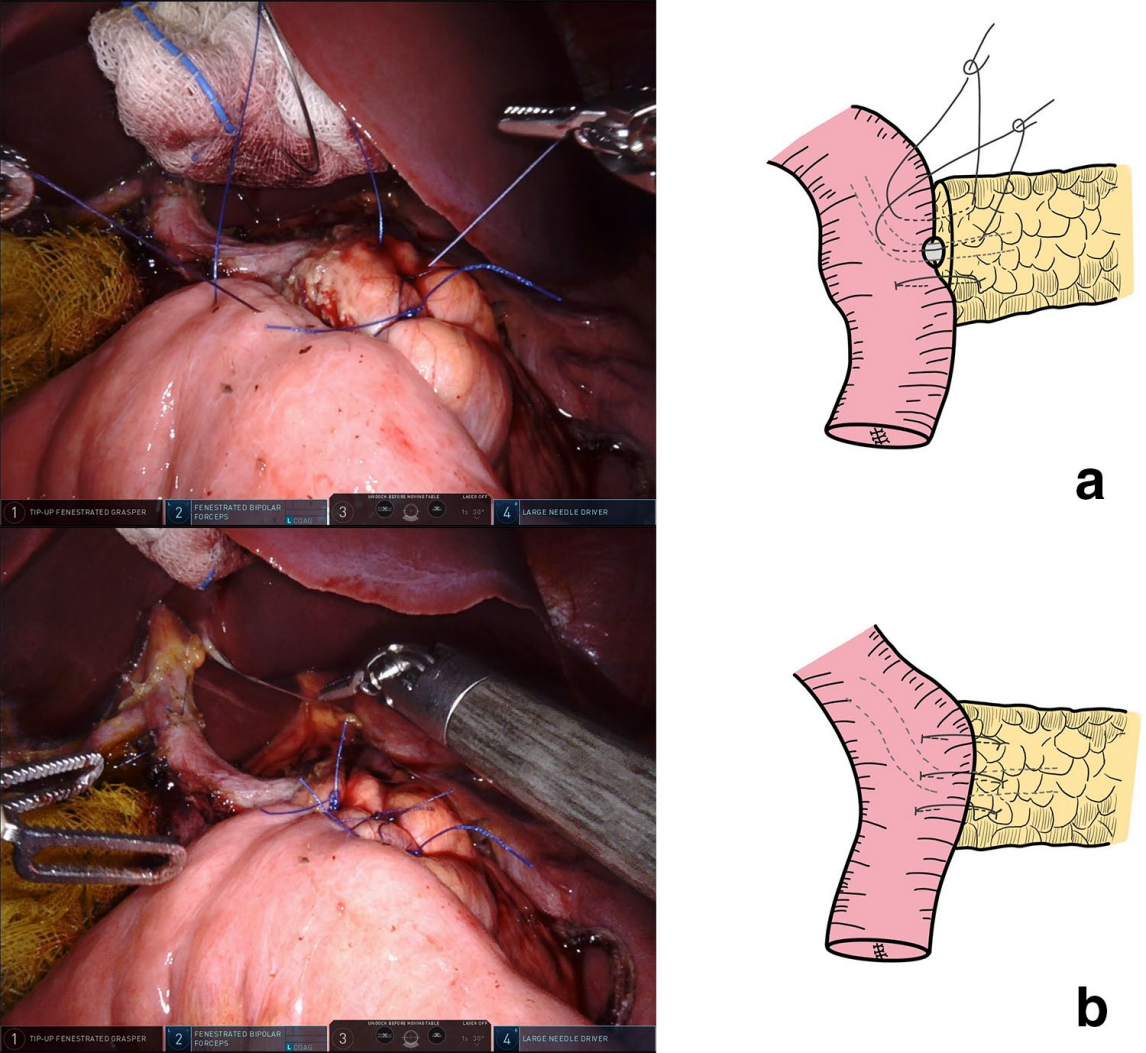
**a** MPD’s anterior wall and jejunal mucosa was anastomosed by reverse order compared to the first duct-to-mucosa stitch. **b** The second duct-to-mucosa stitch and the first penetrating stitch were knotted

Next, to suture the anterior wall of the MPD and jejunal mucosa, the fourth stitch (12 cm in length) was knotted with a 4–0 absorbable Vicryl suture. Subsequently, the first penetrating stitch was knotted (Fig. 6).

**Fig. 7:**
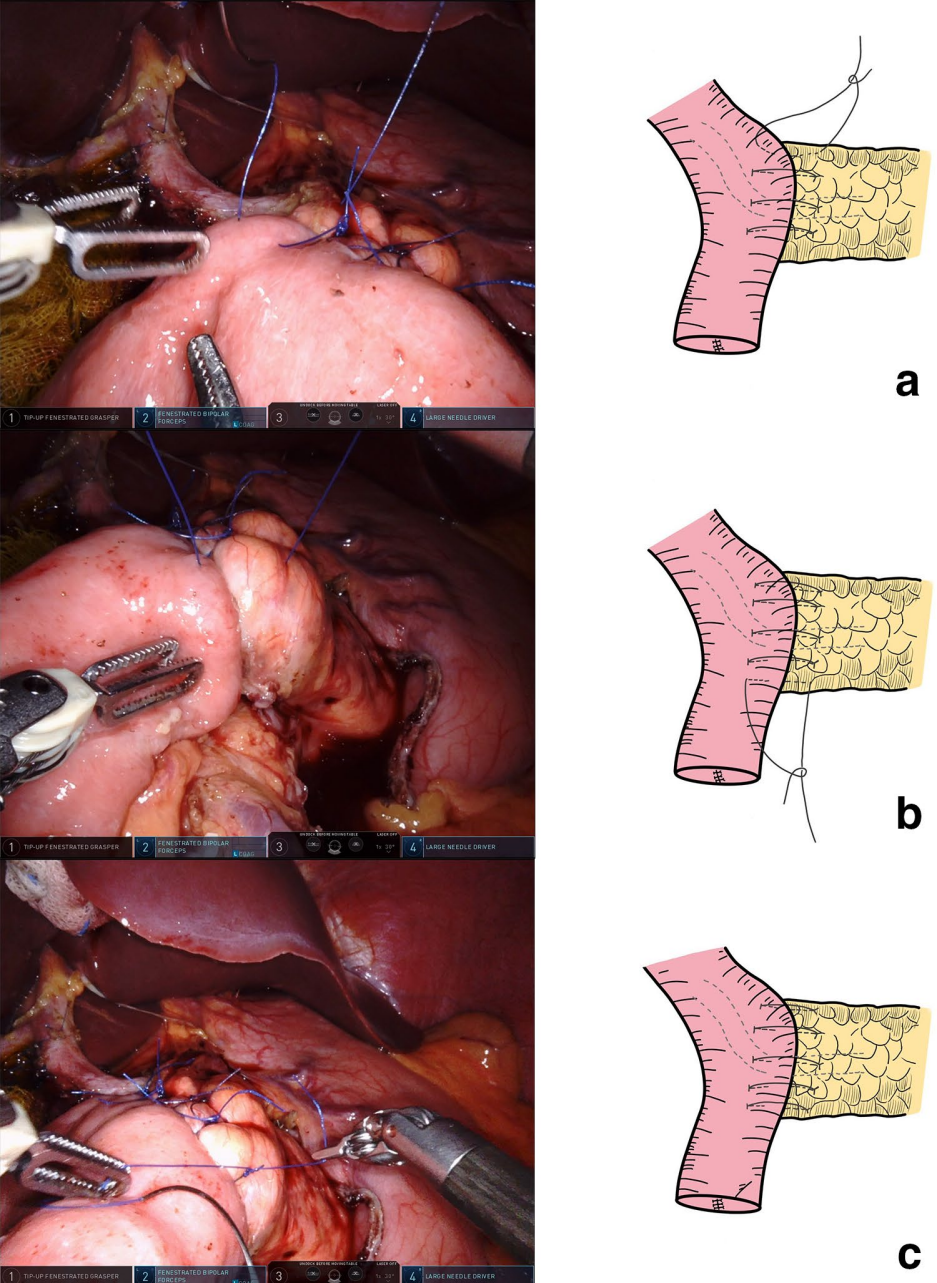
**a, b, c** The last two stitches sutured the upper and lower borders of the pancreatic stump and jejunal wall were performed and knotted

Finally, the last two sutures (12 cm in length), which were used to anastomose the upper and lower borders of the pancreatic stump and jejunal wall, were knotted to compete the anastomosis (Fig. 7).

Further reconstruction for digestive continuity was completed by using the Child procedure, including choledochojejunostomy and gastrojejunostomy. Then, three drainage tubes were placed posterior to the choledochojejunostomy and superior and posterior to the pancreaticojejunostomy.

### Postoperative management

Sandostatin was routinely used after surgery to prevent possible POPF. The amylase concentration of drainage was measured on postoperative days (PODs) 3 and 5, as well as at subsequent time points as necessary. Computed tomography scans (with contrast enhancement) were obtained to assess the fluid collection and guide the management of the drains for each patient on PODs 5–7. For patients with a low-risk of POPF and less than 3000 U/L of amylase, the drainage tube was retreated on POD 3 and then removed on PODs 5–7; for patients with a high-risk of POPF, the time of drainage tube placement needed to be appropriately extended; for patients with POPF, the drains were removed when the total output was < 10 mL for three consecutive days unless fever or infection occurred.

### Statistical analysis

Values are expressed as percentages and medians, according to their distributions. The Mann‒Whitney *U* test was used for non-normally distributed variables, and the chi-square test or Fisher’s exact test was applied to the categorical data. All statistical analyses were performed using SPSS version 25.0 software (SPSS Inc., Chicago, IL, USA). A *P* value of less than 0.05 was considered statistically significant.

## Results

The patients in our study included 57 males and 41 females with a median age of 63 years old (11–77 years old). The median BMI of the patients was 21.43 kg/m^2^ (15.92–32.44 kg/m^2^). Sixty-six patients (66.3%) were diagnosed with jaundice (Table 1).

**Table 1:** Baseline characteristics of the 98 patients who underwent minimally invasive pancreaticoduodenectomy

Patient characteristics		
Age, year, median (range)	63	(11–77)
Male, *n* (%)	57	(58.2%)
Jaundice, *n* (%)	65	(66.3%)
BMI^a^, kg/m^2^, median (range)	21.43	(15.92–32.44)
Past medical history		
Diabetes mellitus, *n* (%)	36	(36.7%)
ASA^b^ score		
I, *n* (%)	4	(4.1%)
II, *n* (%)	76	(77.6%)
III, *n* (%)	17	(17.3%)
IV, *n* (%)	1	(1.0%)
V, *n* (%)	0	(0%)
Pancreatic consistency, *n* (%)		
Soft	44	(44.9%)
Not-soft	54	(55.1%)
Diameter of MPD^c^ ≤ 3 mm, *n* (%)	50	(51.0%)
Diameter of MPD > 3 mm, *n* (%)	48	(49.0%)

^a^*BMI* body mass index; ^b^*ASA* American Society of Anesthesiologists; ^c^*MPD* main pancreatic duct

For the intraoperative situation, the median duration of PD was 350 min (260–480 min), and the median time for performing PJ was 17 min (12–25 min). The median blood loss was 60 mL (10–250 mL), and one patient (1.0%, 1/98) who suffered moderate anemia before the operation received a blood transfusion (Table 2). The pathologic results are listed in Table 3.

**Table 2:** Operative parameters (*n* = 98)

LPD^a^, *n* (%)	75	(76.5%)
RPD^b^, *n* (%)	23	(23.5%)
Operation time, min, median (range)	350	(260–480)
PJ time^c^, min, median (range)	17	(12–25)
Blood loss during operation, mL, median (range)	60	(10–250)
Cases required transfusion, *n* (%)	1	(1.0%)

^a^*LPD* laparoscopic pancreaticoduodenectomy; ^b^*RPD* robotic pancreaticoduodenectomy; ^c^*PJ* time, pancreaticojejunostomy time

**Table 3:** Postoperative pathologic diagnoses (*n* = 98)

Pancreatic head lesion, *n* (%)	37	(37.8%)
Pancreatic ductal adenocarcinoma	21	(21.4%)
IPMN^a^, *n* (%)	4	(4.1%)
pNENs^b^, *n* (%)	3	(3.1%)
SPN^c^, *n* (%)	2	(2.0%)
MCN^d^, *n* (%)	1	(1.0%)
Chronic pancreatitis, *n* (%)	6	(6.1%)
Ampullary adenocarcinoma, *n* (%)	33	(33.7%)
Duodenal adenocarcinoma, *n* (%)	15	(15.3%)
Distal bile duct adenocarcinoma, *n* (%)	11	(11.2%)
Non-neoplastic lesions, *n* (%)	2	(2.0%)

^a^*IPMN* intraductal papillary mucinous neoplasm; ^b^*pNENs* pancreatic neuroendocrine neoplasm; ^c^*SPN* solid pseudopapillary neoplasm; ^d^*MCN* mucinous cystic neoplasm

POPF, based on the international study group on pancreatic fistula definition in 2017 [30], occurred in 4 patients (4.1%). These fistulas were all grade B and were treated successfully by prolonging the time of drainage and percutaneous drain placement (1 patient). Grade A POPFs, which is no longer considered a CR-POPF, were designated as “biochemical leaks” and occurred in 18 patients (18.4%). The median amylase level in the drainage fluid on POD 3 was 87 U/L in all patients. Three patients (3.1%) suffered from an intra-abdominal hemorrhage, and one of them had a right hepatic artery hemorrhage, which was cured by interventional therapy. Another two patients required reoperations; one of them had hemorrhage due to perforation of the jejunal stump, which may have been punctured by the pancreatic duct stenting tube on postoperative day (POD) 24, and the other patient had hemorrhage from the stomach stump on POD 27 due to its perforation. Biliary fistula occurred in 5 patients (5.1%) whose common bile ducts were not dilated. These patients were cured by prolonging the time of drainage and percutaneous drain placement (2 patients). In addition, there were 6 patients (6.1%) with delayed gastric emptying, which resolved after conservative treatment, such as parenteral or enteral nutrition. There were 8 patients (8.2%) with intra-abdominal infection. Of these, three patients were cured by antibiotic therapy with prolonged drainage, and four patients were treated successfully by percutaneous drain placement. One patient died on POD 23 because of severe septic shock that developed from afferent loop obstruction and perforation. Three patients (3.1%) with pulmonary infection were cured by antibiotic therapy. There were no cases of urinary tract infection, and no patients died as a result of POPF. The median postoperative hospital stay was 17 days (9–101 days) (Table 4). It should be stated that Chinese patients usually do not want to be discharged unless their abdominal drains are removed. Therefore, the postoperative hospital stay of our patients was much longer than that in other reports.

**Table 4:** Postoperative complications after pancreaticoduodenectomy (*n* = 98)

Clavien-Dindo class ≥ 3, *n* (%)	7	(7.1%)
POPF^a^	4	(4.1%)
Biochemical leaks, *n* (%)	18	(18.4%)
Grade B, *n* (%)	4	(4.1%)
Grade C, *n* (%)	0	(0%)
Postoperative hemorrhage, *n* (%)	3	(3.1%)
Intra-abdominal hemorrhage, *n* (%)	3	(3.1%)
Upper gastrointestinal bleeding, *n* (%)	0	(0%)
Biliary fistula, *n* (%)	5	(5.1%)
Delayed gastric emptying, *n* (%)	6	(6.1%)
Intra-abdominal infection, *n* (%)	8	(8.2%)
Pulmonary infection, *n* (%)	3	(3.1%)
Urinary tract infection, *n* (%)	0	(0%)
Postoperative days in hospital, d, median (range)	17	(9–101)
Reoperation, *n* (%)	3	(3.1%)
90-day Death, *n* (%)	1	(1.0%)

^a^*POPF* postoperative pancreatic fistula

After grouping according to the diameter of the MPD and the texture of the pancreas, we found that there were no significant differences (*P* > 0.05) in patient characteristics, pathological outcomes, or perioperative situation, including POPF (Tables 5–6). The rates of CR-POPF did not differ significantly (*P* < 0.05) among the four grades through the four-tier classification system according to the ISGPS. The occurrence rates of grade A–D were 3.7%, 3.7%, 4.8%, and 4.4%, respectively (Table 7).

**Table 5:** Comparison between 98 patients with soft and not-soft pancreatic texture

Variable	Soft (*n* = 44)	Not-soft (*n* = 54)	*P*-value
Age, year, median (range)	63.1, (11–77)	60, (30–74)	0.338^a^
Male, *n* (%)	25, (56.8%)	32, (59.3%)	0.807^b^
BMI, kg/m^2^, median (range)	22.00, (16.65–27.13)	21.03, (15.92–32.44)	0.179^a^
Jaundice, *n* (%)	31, (70.5%)	34, (63.0%)	0.435^b^
Diabetes, *n* (%)	16, (36.4%)	20, (37.0%)	0.945^b^
MPD size, *n* (%)			
≤ 3 mm	23, (52.3%)	27, (50%)	0.823^b^
> 3 mm	21, (47.7%)	27, (50%)	0.823^b^
PJ time, min, median (range)	17, (12–25)	17, (12–24)	0.698^a^
Blood loss during operation, ml, median (range)	60, (10–250)	55, (10–200)	0.697^a^
Clavien-Dindo class ≥ 3, *n* (%)	4, (9.1%)	3, (5.6%)	0.697^b^
Postoperative days in hospital, d, median (range)	17, (10–101)	16, (9–49)	0.624^a^
POPF, *n* (%)			
Biochemical leaks	10, (22.7%)	8, (14.8%)	0.314^b^
Grade B	2, (4.5%)	2, (3.7%)	1^b^
Grade C	0, (0%)	0, (0%)	N/A
Intra-abdominal hemorrhage, *n* (%)	2, (4.5%)	1, (1.9%)	0.586^b^
Upper gastrointestinal bleeding, *n* (%)	0, (0%)	0, (0%)	N/A
Biliary fistula, *n* (%)	2, (4.5%)	3, (5.6%)	1^b^
Delayed gastric emptying, *n* (%)	3, (6.8%)	3, (5.6%)	1^b^
Intra-abdominal infection, *n* (%)	4, (9.1%)	4, (7.4%)	1^b^
Pulmonary infection, *n* (%)	1, (2.3%)	2, (3.7%)	1^b^
Urinary tract infection, *n* (%)	0, (0%)	0, (0%)	N/A
Reoperation, *n* (%)	2, (4.5%)	1, (1.9%)	0.586^b^
Death, *n* (%)	0, (0%)	1, (1.9%)	1^c^

^a^Mann–Whitney *U* test, ^b^Chi-square test, ^c^Fisher exact test

**Table 6:** Comparison between 98 patients with large (> 3 mm) and small (≤ 3 mm) MPD

Variable	Large MPD (*n* = 48)	Small MPD (*n* = 50)	*P*-value
Age, year, median (range)	61.9, (35–75)	63, (11–77)	0.994^a^
Male, *n* (%)	31, (64.6%)	26, (52%)	0.207^b^
BMI, kg/m^2^, median (range)	21.82, (17.24–32.44)	21.26, (15.92–31.00)	0.479^a^
Jaundice, *n* (%)	36, (75%)	29, (58%)	0.075^b^
Diabetes, *n* (%)	20, (41.7%)	16, (32%)	0.666^b^
Pancreatic texture, *n* (%)			
Soft	21, (43.8%)	23, (46%)	0.823^b^
Not-soft	27, (56.3%)	27, (54%)	0.823^b^
PJ time, min, median (range)	17, (12–25)	17, (12–23)	0.523^a^
Blood loss during operation, ml, median (range)	60, (10–250)	55, (10–230)	0.886^a^
Clavein-Dindo class ≥ 3, *n* (%)	3, (6.3%)	4, (8%)	1^c^
Postoperative days in hospital, d, median (range)	16, (10–101)	18, (9–51)	0.230^a^
POPF, *n* (%)			
Biochemical leaks	7, (14.6%)	11, (22%)	0.343^b^
Grade B	2, (4.2%)	2, (4%)	1^c^
Grade C	0, (0%)	0, (0%)	N/A
Intra-abdominal hemorrhage, *n* (%)	2, (4.2%)	1, (2%)	0.613^c^
Upper gastrointestinal bleeding, *n* (%)	0, (0%)	0, (0%)	N/A
Biliary fistula, *n* (%)	3, (6.3%)	2, (4%)	0.674^c^
Delayed gastric emptying, *n* (%)	2, (4.2%)	3, (6%)	1^c^
Intra-abdominal infection, *n* (%)	4, (8.3%)	4, (8%)	1^c^
Pulmonary infection, *n* (%)	1, (2.1%)	2, (4%)	1^c^
Urinary tract infection, *n* (%)	0, (0%)	0, (0%)	N/A
Reoperation, *n* (%)	1, (2.1%)	2, (4%)	1^c^
Death, *n* (%)	1, (2.1%)	0, (0%)	0.490^c^

^a^Mann–Whitney *U* test, ^b^Chi-square test, ^c^Fisher exact test

**Table 7:** Clinically relevant postoperative pancreatic fistula for grade A-D anastomoses in 98 patients

Variable	Patients without CR^a^-POPF	Patients with CR^a^-POPF	Rate	*P*-values	
A. Not-soft pancreatic texture and MPD > 3 mm	26	1	3.7%	1^b^	1^b^
B. Not-soft pancreatic texture and MPD ≤ 3 mm	26	1	3.7%		
C. Soft pancreatic texture and MPD > 3 mm	20	1	4.8%	1^b^	
D. Soft pancreatic texture and MPD ≤ 3 mm	22	1	4.4%		
Total	94	4	4.3%	–	–

^a^CR, clinically relevant, ^b^Chi-square test

Based on the selection criteria of the benchmark cases, 27 (27.6%) high-risk (benchmark) and 71 (72.4%) low-risk (non-benchmark) patients constituted the cohort in our study (Table 8). When comparing the postoperative complications between the two groups, the incidence of CR-POPF and intra-abdominal infection in the high-risk group was seemingly higher than that in the low-risk group, but the differences were not statistically significant. The occurrence rates of other complications, such as biliary fistula, DGE, and intra-abdominal hemorrhage, were similar in both groups.

**Table 8:** Comparison of postoperative complications between 98 patients with high-risk (*n* = 27) and low-risk (*n* = 71) patients

Variable	Low-risk patients (*n* = 71)	High-risk patients (*n* = 27)	*P*-values
Clavien-Dindo class ≥ 3, *n* (%)	5, (7.0%)	2, (7.4%)	1^a^
POPF, n (%)			
Biochemical leaks	14, (19.7%)	4, (14.8%)	0.789^a^
Grade B	1, (1.4%)	3, (11.1%)	0.11^a^
Grade C	0, (0%)	0, (0%)	N/A
Intra-abdominal hemorrhage, *n* (%)	2, (2.8%)	1, (3.7%)	1^a^
Upper gastrointestinal bleeding, *n* (%)	0, (0%)	0, (0%)	N/A
Biliary fistula, *n* (%)	4, (5.6%)	1, (3.7%)	1^a^
Delayed gastric emptying, *n* (%)	5, (7.0%)	1, (3.7%)	0.885^a^
Intra-abdominal infection, *n* (%)	4, (5.6%)	4, (14.8%)	0.285^a^
Pulmonary infection, *n* (%)	2, (2.8%)	1, (3.7%)	1^a^
Urinary tract infection, *n* (%)	0, (0%)	0, (0%)	N/A
Reoperation, *n* (%)	2, (2.8%)	1, (3.7%)	1^a^
Death, *n* (%)	1, (1.4%)	0, (0%)	1^b^

^a^Chi-square test, ^b^Fisher exact test

## Discussion

For Kakita PJ anastomosis [24], several sutures should be performed within the MPD, which is rather challenging when the duct is not dilated. According to recent studies, for patients with a small MPD, grade B + C POPF was observed in 6.7% to 19.2% [14, 33, 34], which was troubling to many surgeons.

Initially, we anastomosed the duct to the mucosa by using an interrupted circular suture containing 6 to 8 stitches in OPD. This was theoretically difficult to perform in LPD and RPD due to the narrow operating field view and was prone to cause pancreatic parenchyma lacerations when knotted. The critical change made was to gradually reduce the duct-to-mucosa stitch number from 6 to 8 stitches to 4 stitches, after which anastomosis remained reliable. Finally, we found that just 2 stitches anterior and posterior to the duct were sufficient in OPD. Additional stitches were needed only in the case of an extremely dilating duct. In this series, the maximum MPD was 6 mm, which was also applied to our PJ procedure.

The grade B + C POPF rate (4.1%) was extremely low and much lower than that currently reported in other studies. All POPF cases were cured by simple drainage or abdominal paracentesis. To this end, we analyzed the clinical data of OPDs performed with different PJ techniques in our center from January 2014 to April 2018 and found that the grade B + C POPF rate was 13.5% (11.7% grade B, 1.8% grade C), which was higher than that of our PJ. In summary, reducing the stitch number not only simplified the anastomotic procedure and decreased the difficulty of the operation, especially in LPD and RPD, but also enabled a “duct-to-mucosa” PJ with a penetrating-suture to better suit small-bore MPD patients. To our knowledge, the 2-stitch technique anastomosing the duct and mucosa was much simpler than that used by other surgeons who used penetrating-suture in PD [20, 22, 24].

To further validate the applicability of modified Kakita PJ in anastomoses with different risks, all patients were grouped according to different pancreatic textures and MPD diameters and the four-tier classification system proposed by ISGPS for international comparability [31]. These grouping results showed that, even in cases with a soft pancreas and small MPD, the incidence of CR-POPF was only 4.4%, which indicated that our PJ was effective for cases with different pancreatic textures and MPD diameters.

In addition to the inherent factors of the pancreas, the anastomotic technique, especially the maintenance of blood supply to the PJ, also had a crucial impact on the incidence of POPF. In the conventional duct-to-mucosa or invagination PJ, the pancreatic remnant is mobilized with approximately 0.5–1 cm reserved for the posterior layer suture. This method is not only laborious in some patients, such as in those with chronic pancreatitis, but may also harm the blood supply. Dissociation of the pancreatic remnant was not mandatory in our method, which allowed the needles to puncture from the posterior edge of the pancreatic stump. In addition to protecting the blood supply of the stump, this anastomosis technique is seemingly simplified. This additional unique advantage of our approach may contribute to the reduction in the occurrence of POPF.

For minimally invasive PD, the long learning curve is often difficult for surgeons to overcome. A systematic review showed that 39 cases of LPD and 25 cases of RPD could be recognized as the first stage of the learning curve [35]. According to the cases of minimally invasive PD (42 LPDs and 15 RPDs) performed by the main surgeon before the study combined with the OPD experience of 6 years before the LPD procedure, we thought we had completed the first phase of the learning curve, and the learning period of RPD could be classified as the terminal of phase I. An increasing number of studies have proven that there would be more complex cases in the period of technical competence and challenge [35–37], which could be demonstrated in our study’s pathological results of the high proportion of malignant tumors. However, our study did not report a case of minimally invasive PD combined with blood vessel reconstruction, which was also the goal we would challenge in the future. As for digestive tract reconstruction, PJ tends to be the most difficult step. Simplicity and a short learning curve are required for the optimal PJ technique. Our PJ was performed in OPD at first. After 52 cases of OPD were completed, we started to perform PJ under laparoscopy and then robotically. Although performing PJ under laparoscopy is challenging, we achieved stabilization of the PJ procedure in fewer than 20 cases. Unlike in the three-phase model of the PD learning curve [35–37], the “challenging period” that existed in PD was not suitable for our PJ because once the PJ procedure was established, it could be performed easily in all cases.

Given that some low-volume pancreatic surgery centers do not have much access to cases suitable for minimally invasive PD, it is difficult for them to learn complex PJ procedures. Our PJ, which is simple, safe and effective, has the potential to become the preferred procedure in low-volume pancreatic surgery centers.

However, subject to retrospective study, there were many limitations in our study, including small sample size, single center study and inherent selection bias. The majority of cases (72.4%) were low-risk in our study, and the incidence of CR-POPF in high-risk cases was 11.1%, which indicates that more clinical data on performing PJ in high-risk cases are needed. To further confirm the reliability of this PJ technique, it is necessary to conduct relevant randomized controlled trials in the near future.

## Conclusion

To our knowledge, this modified Kakita PJ procedure is the easiest method to perform using LPD and RPD. This technique is suitable for almost all pancreatic conditions, even in cases with a fragile pancreas stump or small MPD, and it has the potential to become the preferred laparoscopic or robotic PJ procedure in low-volume pancreatic surgery centers.

## Supplementary Information

Below is the link to the electronic supplementary material.

Supplementary file1 (MP4 307902 KB)
